# A Frequency Domain Analysis of the Growth Factor-Driven Extra-Cellular-Regulated Kinase (ERK) Pathway

**DOI:** 10.3390/biology14040374

**Published:** 2025-04-05

**Authors:** Nguyen H. N. Tran, Federico Frascoli, Andrew H. A. Clayton

**Affiliations:** 1 Department of Physics and Astronomy, Optical Sciences Centre, School of Science, Computing and Engineering Technologies, Swinburne University of Technology, Melbourne, VIC 3122, Australia; nt625@drexel.edu; 2 Department of Mechanical Engineering and Mechanics, Drexel University, Philadelphia, PA 19094, USA; 3 Department of Mathematics, School of Science, Computing and Engineering Technologies, Swinburne University of Technology, Melbourne, VIC 3122, Australia; ffrascoli@swin.edu.au

**Keywords:** ERK pathway, EGF, frequency domain, NGF, PC12, transfer function

## Abstract

The ERK pathway is an important biochemical cascade and acts as a master regulator of myriad cell processes including cell proliferation, differentiation, and survival. Early biochemical work established that the timing of ERK phosphorylation was an important determinant of PC12 cell fate, with extended phosphorylation (with nerve growth factor treatment) linked to differentiation but rapid on–off ERK phosphorylation kinetics (with epidermal growth factor treatment) linked to cell proliferation. Recent work from several laboratories has revealed that periodic forcing the phosphorylation of ERK with growth factors, light (optogenetics) or electronically can switch cell fate from proliferative to differentiated depending on type of stimulus (amplitude and frequency). Here, we take an ERK model and analyze it from the frequency domain perspective. The key is the transfer function, which provides a compact description of input (growth factor)–output (ERK activation) behavior over a range of input frequencies, allowing an understanding of system dynamics in terms of amplitude modulations, phase shifts, and signaling bandwidths. Our analysis of transfer functions indicates that, at normal receptor levels, the ERK pathway acts as a negative feedback amplifier to growth factor fluctuations, amplifying them at low receptor occupancy but suppressing them at high receptor occupancy. The frequency dependence is best described as a resonant low pass filter, which selectively filters out high frequency input oscillations. We use the transfer function to predict how different growth factor input dynamics shape ERK activation.

## Simple Summary

Biological signals are not always constant but can oscillate up and down as waves or appear in discrete packets or pulses. How cells process complex input signals from the environment is a key biological question. Here, we take an important biochemical cascade (ERK pathway) and present a frequency domain analysis of its input–output characteristics. Our results show the interesting finding that low average receptor occupancy leads to amplification of input fluctuations, while high receptor occupancy dampens these fluctuations. Our approach provides an alternative view of how biochemical circuits process signals and make predictions that can help explain current experimental results as well as predict new ones.

## 1. Introduction

The extracellular-regulated kinase or ERK pathway is a highly conserved signal transduction pathway across a variety of eukaryotic species, from relatively simple unicellular organisms like yeasts to more complex multicellular organisms like humans [1]. It serves as a master regulator of cell function—including differentiation, proliferation, migration and metabolism [2].

In PC12 cells, a pheochromocytoma derived from rats, the ERK pathway has provided a fascinating model system to examine cell fates. Application of epidermal growth factor to PC12 cells produces transient ERK activation (though the EGF receptor) and cell proliferation while nerve growth factor binds to the TRK receptor and leads to sustained ERK activation and cell differentiation [3].

Recent work by Ryu et al. [4] has revealed that delivering growth factors (i.e., epidermal growth factors) in a series of synthetic periodic pulses of differing frequencies can influence cell fate outcomes, i.e., by re-triggering ERK activation for extended periods. For certain pulse amplitudes and frequencies, a potent differentiated state could be induced with a growth factor that normally is considered to produce a proliferative state only. The authors measured the ERK activation and differentiation extent to pulsed growth factors of different amplitude, frequency, and growth factor type (EGF vs. NGF) and produced an ERK pathway model involving negative and positive feedback loops that accounted for the experimental results.

However, several questions remain from that important study. How do low and medium amplitude EGF pulse trains that differ by a factor of 25 produce similar extents of ERK activation? How does the pathway respond to different types (rectangular, triangular, sinusoidal), amplitudes and frequencies of inputs? Can the model be used to design input sequences with any desired output characteristics, i.e., long ERK decay, oscillatory ERK, short ERK decay, constant ERK level? We hypothesize that a frequency domain analysis can provide a useful framework for addressing these questions.

To address these questions, we undertook a frequency domain analysis of the pathway model presented by Ryu et al. [4]. To perform this analysis, we derived the transfer function [5], which describes how input growth factor sinusoids are demodulated and phase shifted into activated ERK oscillations at different frequencies. The use of transfer functions builds on the previous work of Wiley and Lauffenburger, who used them to construct a transactivation circuit for the EGFR-ERK system [6]. The advantage of this type of analysis is that it enables a characterization of the type of filtering the ERK pathway employs and can be generalized to any type of input signal, whether it be unit impulse, periodic pulses or sinusoids.

The paper is organized as follows. In the Results section, we describe the model of Ryu et al. [4], derive the growth factor to activated ERK transfer function and present Bode plots (i.e., plots of how activated ERK waves are altered in amplitude or phase as a function of input frequency) for different receptor occupancies. We then calculate activated ERK outputs for different pulse types, amplitudes and frequencies.

In the Discussion section, we discuss the relationship between activated ERK outputs and differentiation in the context of a periodically forced input. We discuss how different input shapes may optimize ERK activation characteristic depending on whether differentiation is controlled by the integrated signal, persistence above threshold or modulation.

## 2. Materials and Methods

### 2.1. Dynamical Model of ERK Pathway

The dynamical model of the ERK pathway was taken directly from Ryu et al. (Table 1) [4], specifically Model 1 on page 13 of the appendix. All parameters were fixed in the model. In our paper, we treat EGF and NGF as the inputs in their respective cases and ERK* as the output. To obtain a transfer function from this system of nonlinear first-order differential equations, we begin by defining the following vectors.

$$\mathrm{Input}\;\mathrm{vector}\text{:}\;u=\left[EGF\right]=\left[u_{1}\right]\mathrm{or}\;u=\left[NGF\right]=\left[u_{1}\right]$$

$$\mathrm{State}\;\mathrm{vector}\text{:}\;x=\left[\begin{array}{c} R\\ R^{*}\\ {Ras}^{*}\\ {\;\mathrm{Ras}}^{*}\\ {Raf}^{*}\\ {Raf}^{*}\\ MEK\\ {\;\mathrm{MEK}}^{*}\\ ERK\\ ERK*\\ NFB\\ NFB*\\ PFB\\ PFB*\\ dusp\\ DUSP \end{array}\right]=\left[\begin{array}{l} x_{1}\\ x_{2}\\ x_{3}\\ x_{4}\\ x_{5}\\ x_{6}\\ x_{7}\\ x_{8}\\ x_{9}\\ x_{10}\\ x_{11}\\ x_{12}\\ x_{13}\\ x_{14}\\ x_{15}\\ x_{16} \end{array}\right]\;\;\;\;\mathrm{Dynamics}\;\mathrm{vector}\text{:}\;F=\frac{d}{dt}\left[\begin{array}{c} R\\ R^{*}\\ {Ras}^{*}\\ {\;\mathrm{Ras}}^{*}\\ {Raf}^{*}\\ {Raf}^{*}\\ MEK\\ {\;\mathrm{MEK}}^{*}\\ ERK\\ ERK*\\ NFB\\ NFB*\\ PFB\\ PFB*\\ dusp\\ DUSP \end{array}\right]=\left[\begin{array}{l} -v_{1}\\ +v_{1}\\ -v_{6}\\ +v_{6}\\ -v_{5}-v_{5a}\\ +v_{5}+v_{5a}\\ -v_{4}\\ +v_{4}\\ -v_{2}\\ +v_{2}\\ -v_{3a}\\ +v_{3a}\\ -v_{7a}\\ +v_{7a}\\ +v_{8}-v_{9}\\ +v_{10}-v_{11} \end{array}\right]=\left[\begin{array}{c} F_{1}\\ F_{2}\\ F_{3}\\ F_{4}\\ F_{5}\\ F_{6}\\ F_{7}\\ F_{8}\\ F_{9}\\ F_{10}\\ F_{11}\\ F_{12}\\ F_{13}\\ F_{14}\\ F_{15}\\ F_{16} \end{array}\right]$$

$$\mathrm{Output}\;\mathrm{vector}\text{:}\;y=\left[{ERK}^{*}\right]=\left[y_{1}\right]\;\;\;\;\;\;\;\mathrm{Observation}\;\mathrm{vector}\text{:}\;G=\left[{ERK}^{*}\right]=\left[G_{1}\right]$$

where the $v_{n}$ terms come from the appendix of [4] as follows:

$$v_{1}=k1R\times R\times u1-kd1R\times PtaseR\times R^{*}$$

$$v_{6}=k6R\times R^{*}\times \frac{Ras}{K6+Ras}-kd6\times GAP\times \frac{Ras^{*}}{D6+Ras^{*}}$$

$$v_{5}=k5\times Ras^{*}\times \frac{Raf}{K5+Raf}*\frac{KNFB^{2}}{KNFB^{2}+{\left(NFB^{*}\right)}^{2}}-kd5\times PtaseRaf\times \frac{Raf^{*}}{D5+Raf^{*}}$$

$$v_{5a}=kPFB\times PFB^{*}\times \frac{Raf}{KPFB+Raf}$$

$$v_{4}=k4\times Raf^{*}\times \frac{MEK}{K4+MEK}-kd4\times PtaseMEK\times \frac{MEK^{*}}{D4+MEK}$$

$$v_{2}=k2\times MEK^{*}\times \frac{ERK}{K2+ERK}-kd2\times DUSP\times \frac{ERK^{*}}{D2+ERK^{*}}$$

$$v_{3a}=k3F\times ERK^{*}\times \frac{NFB}{K3+NFB}\times \frac{{\left(R^{*}\right)}^{2}}{K3R^{2}+{\left(R^{*}\right)}^{2}}-kd3\times PtaseNFB\times \frac{NFB^{*}}{D3+NFB^{*}}$$

$$v_{7a}=k7\times ERK^{*}\times R^{*}\times \frac{PFB}{K7+PFB}-kd7\times PtasePFB\times \frac{PFB^{*}}{D7+PFB^{*}}$$

$$v_{8}=duspbasal\times \left(1+duspind\times \frac{{\left(ERK^{*}\right)}^{2}}{Kdusp+{\left(ERK^{*}\right)}^{2}}\;\right)\times \frac{log\left(2\right)}{Tdusp}$$

$$v_{9}=dusp\times \frac{log\left(2\right)}{Tdusp}$$

$$v_{10}=dusp\times \frac{log\left(2\right)}{TDUSP}$$

$$v_{11}=DUSP\times \frac{log\left(2\right)}{TDUSP}$$

The same goes for the associated constants.

For examples of how these are set up in MATLAB (version number R2023a), please see the following links:

1. Implementation of symbolic dynamical system equations: https://github.com/nguyenhntran/ERKpaper2025/blob/main/SourceCode/ERKpathway_EGFactivation/General_TransFunc/src/PART1.m (accessed on 2 April 2025).
2. Implementation of EGF constants: https://github.com/nguyenhntran/ERKpaper2025/blob/main/SourceCode/ERKpathway_EGFactivation/RectangularPulseTrain/TransferFunction/src/PART2.m (accessed on 2 April 2025).
3. Implementation of NGF constants: https://github.com/nguyenhntran/ERKpaper2025/blob/main/SourceCode/ERKpathway_NGFactivation/RectangularPulseTrain/Equilibria/src/Prelim.m (accessed on 2 April 2025).

At equilibrium, we denote the steady-state values of state and input variables as

$$\left(x_{e},u_{e}\right)=\left(x_{1e},x_{2e},x_{3e},x_{4e},x_{5e},x_{6e},x_{7e},x_{8e},x_{9e},x_{10e},x_{11e},x_{12e},x_{13e},x_{14e},x_{15e},x_{16e},u_{1e}\right)\;.$$

To analyze the system’s local dynamics behavior around this equilibrium, we compute the four **Jacobian matrices**: **A** (state matrix), **B** (input matrix), **C** (output matrix) and **D** (feedthrough matrix). These matrices are defined at each row r and column c as follows:

$$A_{rc}={\left.\frac{\partial F_{r}}{\partial x_{c}}\right|}_{\left(x_{e},u_{e}\right)}B_{rc}={\left.\frac{\partial F_{r}}{\partial u_{c}}\right|}_{\left(x_{e},u_{e}\right)}C_{rc}={\left.\frac{\partial G_{r}}{\partial x_{c}}\right|}_{\left(x_{e},u_{e}\right)}D_{rc}={\left.\frac{\partial G_{r}}{\partial u_{c}}\right|}_{\left(x_{e},u_{e}\right)}\;.$$

The transfer function simply follows from substituting these matrices into

$$T(s)=C(sI-A{)}^{-1}B+D.$$

### 2.2. Non-Sinusoidal Periodic Forcing Simulations

A periodic waveform of arbitrary shape f(t) and period T can be decomposed into a set of sinusoids about a mean as follows:

$$f\left(t\right)=\frac{a_{0}}{2}+\sum_{n=1}^{N}\;a_{n}\cos\left(\frac{2\pi }{T}nt\right)+\sum_{n=1}^{N}\;b_{n}\sin\left(\frac{2\pi }{T}nt\right),$$

where

$$a_{0}=\frac{2}{T}\int_{0}^{T}\;f\left(t\right)dt\;,\;\;a_{n}=\frac{2}{T}\int_{0}^{T}\;f\left(t\right)\cos\frac{2\pi nt}{T}dt\;,\;\;b_{n}=\frac{2}{T}\int_{0}^{T}\;f\left(t\right)\sin\frac{2\pi nt}{T}dt.$$

For a transfer function with frequency response functions M(ω) and ϕ(ω), the sinusoidal terms are transformed as follows:

$$\begin{array}{c} \sum_{n=1}^{N}\;a_{n}\cos\left(\frac{2\pi }{T}nt\right)\to \sum_{n=1}^{N}\;M\left(\omega_{n}\right)a_{n}\cos\left(\frac{2\pi }{T}nt+\phi \left(\omega_{n}\right)\right)\\ \sum_{n=1}^{N}\;b_{n}\sin\left(\frac{2\pi }{T}nt\right)\to \sum_{n=1}^{N}\;M\left(\omega_{n}\right)b_{n}\sin\left(\frac{2\pi }{T}nt+\phi \left(\omega_{n}\right)\right) \end{array}$$

The mean term of the input, $a_{0}/2$, is transformed into the equilibrium output of the dynamical system when driven by a constant input of $a_{0}/2$ over a long period. This holds under the assumption that the sinusoidal amplitudes are sufficiently small. Denoting this equilibrium output as $F^{\prime }\left(t\right)$, we have

$$\frac{a_{0}}{2}\to F^{\prime }\left(t\right)\;.$$

The transformation of *f(t)* due to a transfer function is, therefore,

$$F(t)=F^{\prime }(t)+\sum_{n=1}^{N}\;M\left(\omega_{n}\right)a_{n}\cos\left(\frac{2\pi }{T}nt+\phi \left(\omega_{n}\right)\right)+\sum_{n=1}^{N}\;M\left(\omega_{n}\right)b_{n}\sin\left(\frac{2\pi }{T}nt+\phi \left(\omega_{n}\right)\right)$$

For a rectangular pulse train, the Fourier decomposed input takes the form

$$f\left(t\right)=A\frac{\tau }{T}+\sum_{n=1}^{\infty }\;\left[\frac{A}{\pi n}\sin\left(2\pi n\frac{\tau }{T}\right)\right]\cos\left(2\pi n\frac{t}{T}\right)+\sum_{n=1}^{\infty }\;\left[\frac{A}{\pi n}\left(1-\cos\left(2\pi n\frac{\tau }{T}\right)\right)\right]\sin\left(2\pi n\frac{t}{T}\right)$$

The first term in the expansion, Aτ/T, is the mean about which the sinusoids in the latter two terms oscillate. This Fourier description implies that square wave pulsing is effectively a multi-frequency periodic forcing experiment of sinusoids about the mean Aτ/T. Its corresponding output has the form

$$\begin{array}{l} F(t)=F^{\prime }(t)+\sum_{n=1}^{\infty }\;M\&\left(\frac{2\pi n}{T}\right)\left[\frac{A}{\pi n}\sin\left(2\pi n\frac{\tau }{T}\right)\right]\cos\left(2\pi n\frac{t}{T}+\phi \left(\frac{2\pi n}{T}\right)\right)\\ \;\;\;\;\;\;\;\;\;+\sum_{n=1}^{\infty }\;M\left(\frac{2\pi n}{T}\right)\left[\frac{A}{\pi n}\left(1-\cos\left(2\pi n\frac{\tau }{T}\right)\right)\right]\sin\left(2\pi n\frac{t}{T}+\phi \left(\frac{2\pi n}{T}\right)\right). \end{array}$$

For a triangular pulse train, the Fourier decomposed input takes the form

$$f(t)=\frac{A}{2}+\sum_{n=1}^{\infty }\;\left[-\frac{4A}{(2n-1{)}^{2}\pi^{2}}\right]\cos\left(\left(2n-1\right)2\pi \frac{t}{T}\right)$$

Likewise, the corresponding output has the form

$$F\left(t\right)=F^{\prime }\left(t\right)+\sum_{n=1}^{\infty }\;M\left(\frac{\left(2n-1\right)2\pi }{T}\right)\left[-\frac{4A}{(2n-1{)}^{2}\pi^{2}}\right]\cos\left(2\pi n\frac{t}{T}+\phi \left(\frac{\left(2n-1\right)2\pi }{T}\right)\right)$$

### 2.3. Software

All calculations, simulations and plots were performed using MATLAB (version number R2023a), including the Control Systems Toolbox.

### 2.4. Model Validation

**Figure 1:**
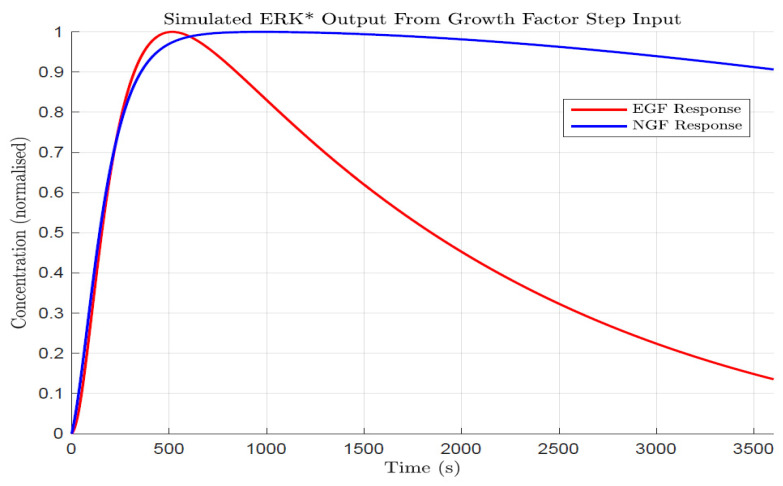
Simulated kinetics of ERK activation (ERK*) following stimulation with either EGF (red line) or NGF (blue line). The results reveal that EGF triggered ERK activation is transient whilst NGF-triggered ERK is sustained. Note that the concentration of activated ERK is expressed in normalized units (fraction activation) to compare with Ref. [4].

To validate our model, we need some means to compare theory with experiment. As far as we are aware, there are no experimentally determined transfer functions for EGF or NGF stimulated ERK activation in pheochromocytoma PC12 cells with which we can compare our theoretical model. However, published kinetics of ERK activation following step-inputs of EGF or NGF are (readily) available [4]. We therefore used the derived transfer functions to calculate the ERK activation kinetics following a step input of EGF or NGF. As anticipated from previous experimental studies, ERK activation following EGF stimulation is transient, while ERK activation following NGF stimulation is sustained. More specifically, as shown in Figure 1 following EGF stimulation, the initial ERK activation is zero and then increases in time, reaching a maximum around 5–10 min, and then decreases over time, reaching about 10–20% of the peak value in 1 h. For NGF, ERK activation also increases to a maximum value after 5–10 min but then decreases more slowly, reaching 80–90% of the peak value at 1 h. These results agree well with the Pertz lab experimental results [4] and other experimental observations of the PC12 pheochromocytoma cells.

## 3. Results

**Figure 2:**
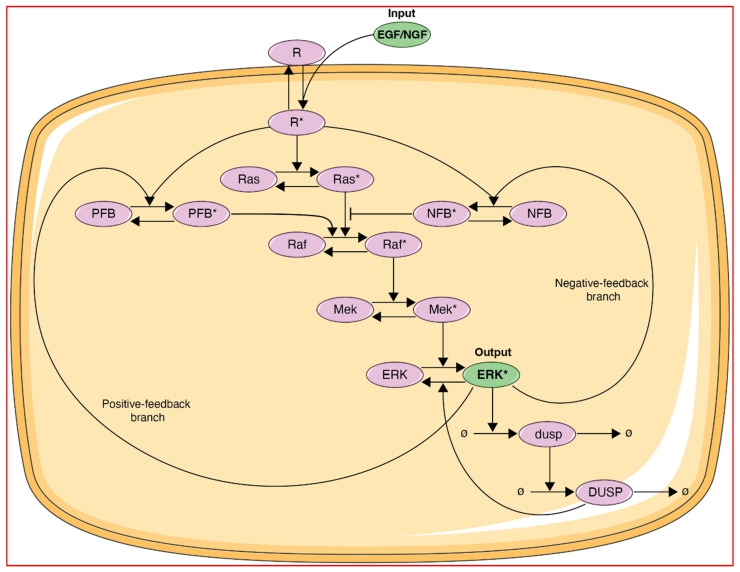
Schematic representation of a growth factor-triggered extra-cellular-regulated kinase (ERK) pathway model [4]. Bubbles denote growth factors (EGF or NFG), receptors (R = EGFR (with EGF) or TrkA (with NGF) and major ERK pathway components (Ras, Raf, Mek, ERK, DUSP), and unidentified positive or negative feedback proteins (PFB or NFB). Asterix signifies activated (phosphorylated) protein. The figure includes adaptation of emptycell-1 icon by Servier (CC-BY 3.0, smart.servier.com).

The ERK pathway model described by Ryu et al. [4] is represented in Figure 2. The input in this model is the ligand (EGF or NGF) which binds to its cognate receptor (R = EGFR or Trk), leading to receptor activation (R*). The receptor activation is then propagated via the core Ras-Raf-MEK signaling cascade as a series of activation/inactivation cycles leading to the output in this model, which is activated ERK. In addition, DUSP and either negative feedback proteins (for EGF- and NGF-driven processes) or positive feedback proteins (for NGF-driven processes) are included to provide negative feedback (for EGF and NGF) and positive feedback (for NGF only). The set of differential equations and parameters pertaining to this model are in Ref. [4] and also in the following link: http://github.com/nguyenhntran/ERKpaper2025 (accessed on 2 April 2025).

To derive the transfer function of this pathway, the differential equations were converted into Jacobian matrices and Laplace space (see Section 2). The transfer functions for different initial conditions were represented as a ratio of polynomials in the complex frequency s, Equation (1).

(1) $$T_{EGF\to ERK*}(s)=\frac{a_{1}s^{3}+a_{2}s^{2}+a_{3}s^{1}+a_{4}}{b_{1}s^{8}+b_{2}s^{7}+b_{3}s^{6}+b_{4}s^{5}+b_{5}s^{4}+b_{6}s^{3}+b_{7}s^{2}+b_{8}s^{1}+b_{9}}$$

**Figure 3:**
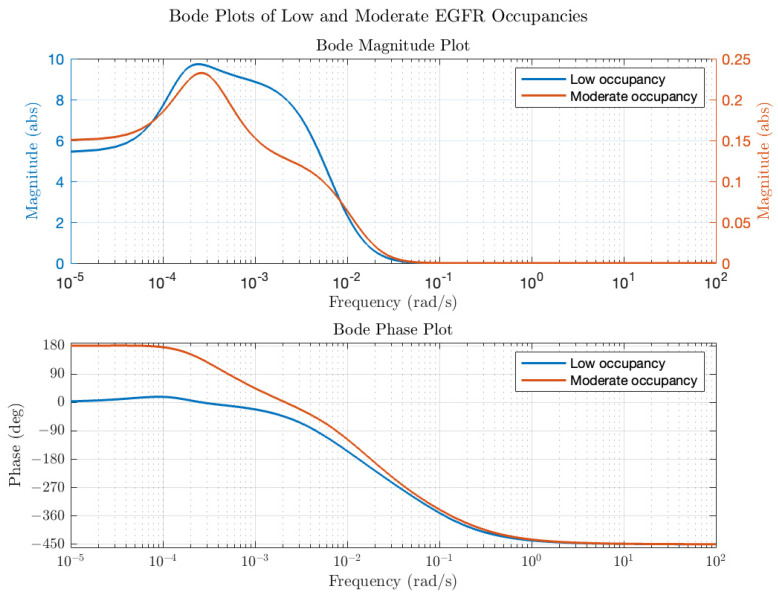
EGF-ERK Bode plots as a function of average receptor occupancy. Low occupancy, corresponding to a mean EGF input of 0.01 Kd, is plotted in blue. Medium receptor occupancy, corresponding to a mean EGF input of 0.48 Kd, is plotted in red.

**Figure 4:**
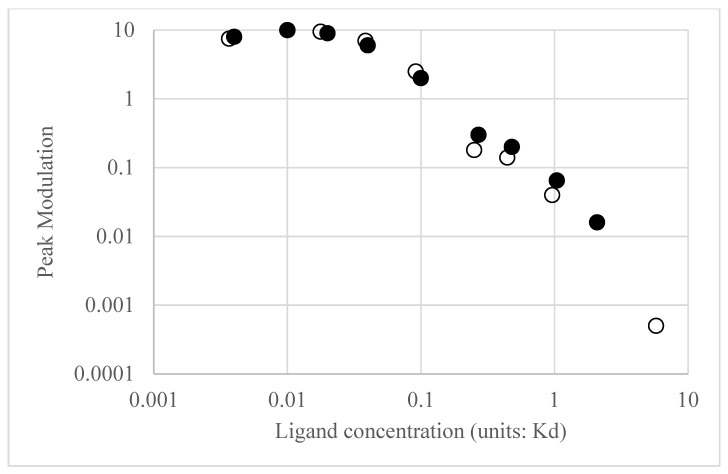

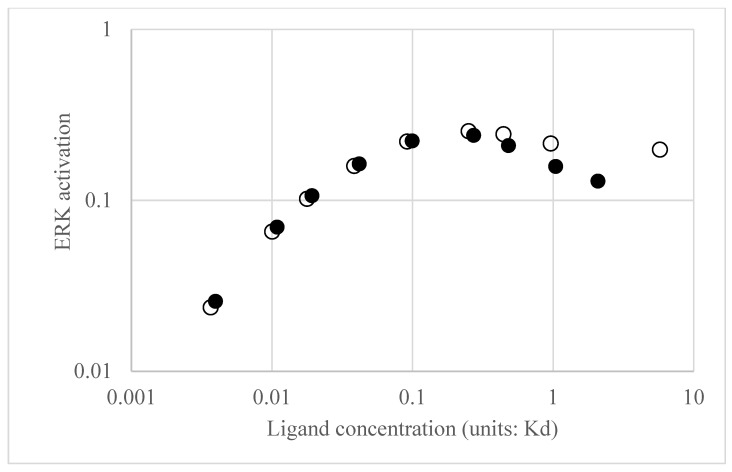
**Upper plot**: Plot of maximal modulation as a function of EGF (filled symbols) or NGF concentration (unfilled symbols). **Lower plot**: Plot of equilibrium ERK activation as a function of EGF (filled symbols) or NGF (unfilled symbols) concentration.

The constants in Equation (1) (a_1_ to a_4_ and b_1_ to b_4_) depend on the initial conditions in the model (ligand concentration, concentrations of components and rate parameters). Of note is that while the model contains 19 different species and 38 rate parameters, the transfer function only contains 13 constants and provides a compact description of input (EGF) to (ERK) dynamics. The transfer functions were then converted into modulation and phase as a function of average receptor occupancy and ligand concentration input frequency. Typical Bode plots for low and moderate EGFR occupancy are in Figure 3. At low average receptor occupancy (Figure 3, blue line), corresponding to the lower end of physiological concentration of EGF ([EGF] = 0.01 K_d_ = 0.02 nM), the Bode magnitude plot reveals a gain of a factor of 5 or more in the frequency range of 10^−5^–10^−2^ rad/s and a decrease in gain beyond 10^−2^ rad/s approaching <<1 as the frequency increases. The dependence of modulation on frequency resembles a typical low pass filter; in other words, input oscillations of low frequency are passed (and amplified) by the ERK circuit but high frequency oscillations are effectively dampened. The phase as a function of frequency also reveals interesting behavior. At low frequencies, the phase delay of the output is zero, meaning that ERK activation oscillations are in-phase with the input EGF oscillations. However, as the frequency of the input EGF oscillations increases, there is a phase delay between the input and output oscillations. The total phase difference of 450 degrees is consistent with a linear kinetic chain comprising 5 components (each component in a linear chain contributes a 90-degree phase difference). Thus, at this low level of input, the system behaves as if the negative regulators of activation (DUSP and the negative feedback loop) are not affecting the dynamics; in other words, negative feedback is at a low level. At the upper end of the EGF physiological range ([EGF] = 0.5 K_d_ = 1 nM), the calculated Bode plot (Figure 3, red line) is distinctly different from that of Figure 3. First, at all frequencies in the range of 10^−5^–10^−2^ rad/s the modulation is less than 1, which implies that the signaling circuit is suppressing input fluctuations. Second, the phase plot (Figure 3, red line) shows that at low frequencies, the output oscillations are 180 degrees out of phase with the input oscillations. In other words, as the input concentration exceeds the equilibrium value, the output ERK activation level will decrease relative to the equilibrium value and vice versa. Thus, the system is acting as a negative feedback system, akin to how when a Hookean spring is compressed or extended, it exerts a force in the opposite direction to resist displacements to its equilibrium position. Bode plots for other receptor occupancies were also computed (not all shown for the sake of brevity) and show the trend that as average receptor occupancy increases, the Bode magnitude peak overall decreases. A plot of the peak modulation as a function of EGF concentration is shown in Figure 4. The transition from amplifier to suppressor of input oscillations appears to occur between 0.2 and 0.5 EGF K_d_ = 0.4–1 nM EGF, which represents the high end of physiological EGF concentrations. While the modulation is important in assessing how the circuit amplifies or suppresses sinusoidal fluctuations about the average value, it is also important to consider the equilibrium ERK activation state as a function of EGF concentration. Figure 4 reveals the calculated ERK activation level as a function of EGF. As expected, as EGF concentration increases, the ERK activation also increases. However, the ERK activation reaches a plateau and then begins to slightly decrease with EGF concentration. As noted previously [6], this is the expected behavior of a negative feedback amplifier, where the feedback is enhanced with increasing input.

**Figure 5:**
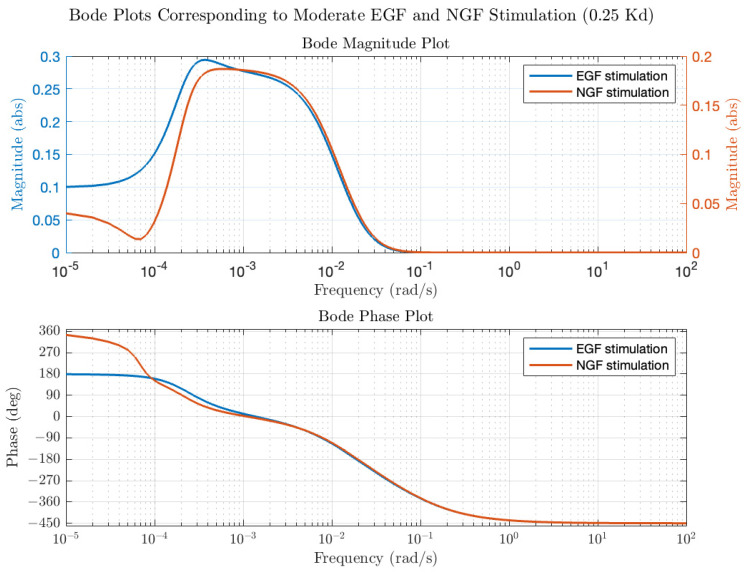
EGF-ERK and NGF-ERK Bode plots at moderate levels of EGF and NGF stimulation (0.25 Kd), illustrated in blue and red, respectively.

Transfer functions and Bode plots were also calculated for NGF stimulation. Qualitatively, the plots at low and high average receptor occupancy were like the EGF plots and the trends in modulation and average ERK activation were similar for concentrations of NGF that produce low receptor occupancy (see Figure 4, open circles for NGF stimulation). However, at higher NGF concentrations, the ERK activation level plateaued but, unlike EGF, remained constant. Differences in the Bode plots were observed for moderate ([L] = 0.25 K_d_) NGF and EGF stimulation, as shown in Figure 5, particularly in the phase behavior. For EGF, the low frequency phase was 180 degrees, increasing to −450 degrees at the high frequencies. For NGF, the low frequency phase was 360 degrees, increasing to degrees at higher frequencies. The total change in phase was 630 degrees for EGF and 810 degrees for NGF. The larger phase for the NGF-triggered circuit implies that additional molecules are involved in shaping the input (relative to those involved in EGF), as expected. We next use the transfer functions to calculate ERK activation responses to periodic inputs.

The transfer function approach allows us to compute the output to any periodic input, since any input can be presented as a sum of sine waves of differing frequency and the transfer function provides the necessary phase and modulation of the output to each input frequency. We use the Fourier principle to calculate the ERK activation kinetics to rectangular pulses of EGF (like the experiments of Pertz [4]) and triangular pulses of EGF (similar to the experiments of Ningsih [7]). Here, we present some representative examples of the simulations.

**Figure 6:**
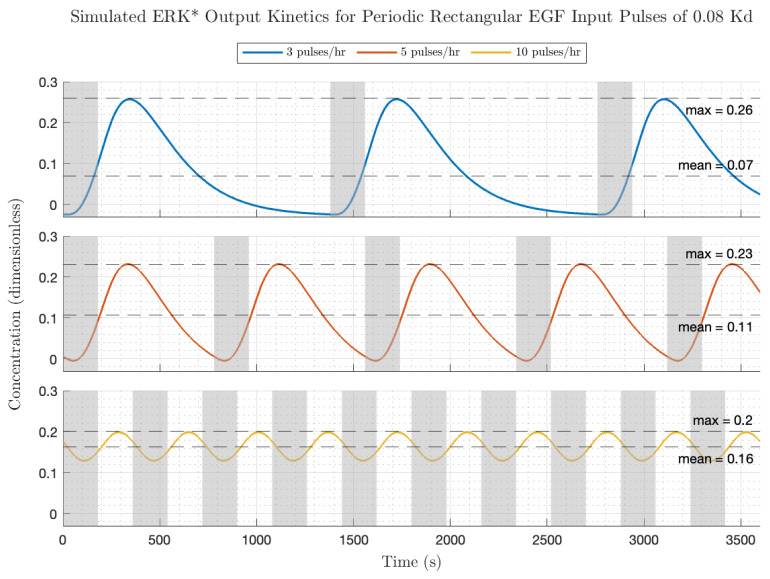
Simulated ERK activation (ERK*) kinetics to periodic rectangular pulses of EGF at low amplitude (0.08 Kd). Gray shaded boxes indicate windows of time during which the EGF pulses are on. **Top**, **middle** and **bottom** panels have identical pulse durations of 180 s (3 min), but different pause durations of 1200 s (20 min), 600 s (10 min) and 360 s (3 min), respectively.

Figure 6 depicts the simulated EGF input and ERK* output kinetics for periodic rectangular pulses (pulse amplitude = 0.08 Kd; pulse duration 3 min) at different pulse frequencies (upper—3 pulses/h; middle—5 pulses/h; lower—10 pulses/h). Increasing the frequency of fixed width EGF pulses from low, middle, to high increased the average EGF dose (0.01, 0.02, 0.04 Kd) and increased the equilibrium ERK* level (0.07, 0.11, to 0.16 ERK*). However, because of the properties of the transfer function, the relative modulation decreased with increasing pulse frequency. A rough figure of merit for the gain modulation is the peak amplitude divided by the equilibrium ERK level, which equaled 3.7, 2.2 and 1.2, for the low, medium and high frequencies. These two factors can account for the small decrease in ERK activation transient peaks with increasing frequency (frequency = low, middle, high; ERK* = 0.26, 0.23, 0.2). The ERK activation pulse profiles were also somewhat dependent on pulse frequency. At low EGF pulse frequencies (Figure 5 top), after each EGF pulse, the ERK* activity increases from a zero value to a finite peak value after some time delay and then decreases to a value close to zero. Qualitatively, the transient increase then decrease in ERK* resembles the low single pulse frequency experiments carried out by the Pertz lab [4]. In the context of the frequency domain, the smearing out of the rectangular input pulse in the ERK* output occurs because the harmonics in the input are phase-delayed due to the frequency-dependent phase in the transfer function. Increasing the frequency produces a more sinusoidal-like output with a lower relative fluctuation compared to the low frequency above. Pushing the fundamental pulse frequency higher puts the input into a part of the Bode plot where there is a rapid roll off in modulation. This essentially means that the higher harmonics from the rectangular pulse are almost completely suppressed, leaving only the fundamental frequency sinusoid as the major contribution.

**Figure 7:**
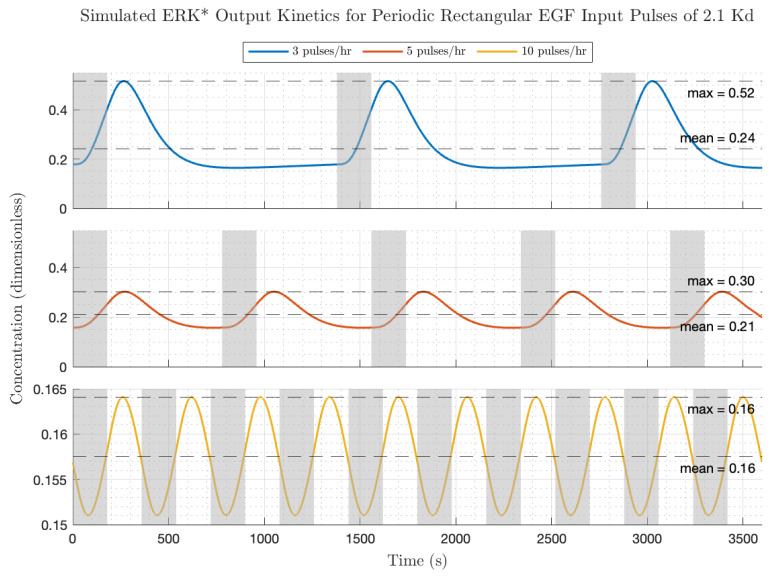
Simulated ERK activation (ERK*) kinetics to periodic rectangular pulses of EGF at high amplitude (2.1 Kd). Gray shaded boxes indicate windows of time during which the 180 s EGF pulses are on. Respective pause durations for the **top**, **middle** and **bottom** panels are 1200 s (20 min), 600 s (10 min) and 360 s (3 min).

The influence of increasing the EGF input pulse amplitude by a factor of 25 on the ERK* activation dynamics is depicted in Figure 7 (all other conditions are shown in Figure 6). Remarkably, the peak activation levels of ERK* are within a factor of 2 of the ERK* low amplitude pulses (frequency = low, medium, high; ERK = 0.52, 0.30, 0.16). Because of the larger receptor occupancy, the equilibrium ERK* values were higher (frequency = low, medium, high; ERK* equilibrium = 0.24, 0.21, 0.16) but the gain modulation values (peak/mean) were lower (frequency = low, medium, high; gain modulation = 2.16, 1.44, 1.04). Interestingly, the transient kinetics of the ERK* following low frequency, high amplitude EGF pulsing appears to be faster than for the low amplitude case in Figure 5. Because the influence of negative feedback is apparent at this level of receptor occupancy in the ERK* versus EGF and also the peak modulation as a function of EGF plots, and in the appearance of a 180-degree phase shift at low frequencies, we suggest that the increased feedback level is responsible for the rapidity of the on–off kinetics to the low frequency, high-amplitude EGF pulses. The behavior of the circuit in response to rectangular inputs is therefore a trade-off between increased average ERK activation, which increases with increased average EGF dose (proportional to pulse frequency and amplitude per pulse), and modulation about the average ERK activation, which decreases with increased pulse frequency and amplitude pulse. We next consider the circuit responses to triangular (almost sinusoidal) periodic forcing with EGF.

**Figure 8:**
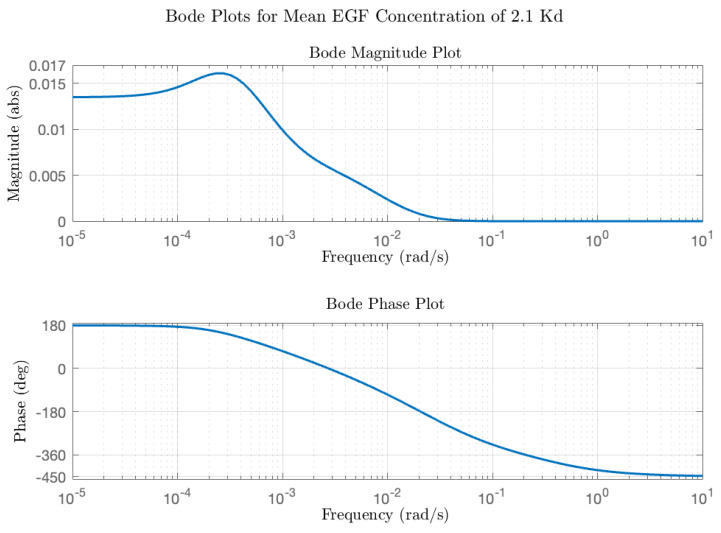
EGF-ERK Bode plot for average EGF concentration of 2.1 K_d_.

**Figure 9:**
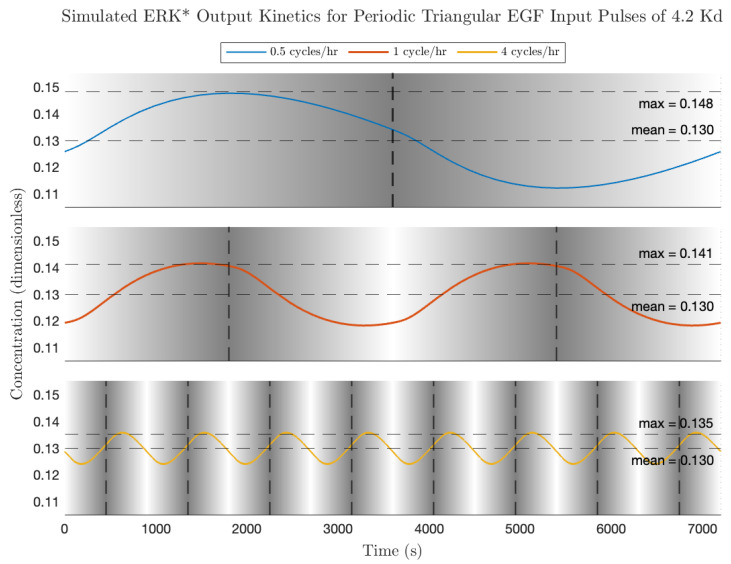
Simulated ERK activation (ERK*) dynamics to periodic triangular pulses of EGF at high amplitude (4.2 Kd). Background shading indicates EGF stimulation concentration, ranging from 0 Kd (white) to 4.2 Kd (gray). Vertical dashed lines indicate times at which EGF stimulation is maximal. Gradient increases linearly from white to gray.

The Bode modulation and phase plot is shown in Figure 8, calculated for the average EGF concentration employed in the experiments of Ningsih et al. [7] ([EGF] = 2.1 Kd). As expected for this high (non-physiological concentration) ligand concentration, the modulations at all frequencies are less than 1, and the phase difference at low frequency is close to 180 degrees. Simulated plots of activated ERK dynamics in response to triangular pulse trains of increasing frequency are shown in Figure 9. Of note is that the average ERK activity is identical for all frequencies; the effect of increasing the pulse frequency is to decrease the modulation of ERK* about the equilibrium value. This can be understood from the Bode magnitude plot, which shows a decreased modulation from 0.5 cycles/h (8.7 × 10^−4^ rad/s), 1 cycle/h (1.7 × 10^−3^ rad/s), through to 4 cycles/h (7 × 10^−3^ rad/s). As for the rectangular pulse experiments, the signaling circuit has a measurable impact on the shape of the output ERK* pulses. High frequency features in the input are effectively blurred out by the signaling circuit-at the frequencies simulated here, all of the higher-order harmonics are located in the part of the transfer function where the modulation is rapidly decreasing, i.e., the modulation becomes proportional some powers of 1/s. In this region, the signaling circuit is effectively integrating (averaging) the high-frequency components. We next discuss responses to sinusoidal inputs and make a prediction regarding conditions for maximal ERK activation.

As far as we are aware, there have been no experiments looking at pure sinusoidal-shaped EGF inputs. The output from a sinusoidal input will be a sine wave with identical frequency but demodulated and phase shifted. The DC value for the ERK activation will be dependent on the average receptor occupancy and EGF dose (Figure 4). The modulation and phase will depend on the frequency and the average EGF dose (Figure 3 and Figure 4). From these considerations, we would predict that an EGF sinewave with average EGF concentration of between 0.02 and 0.04 Kd, and frequency of 2.5 × 10^−4^–3 × 10^−3^ rad/s (i.e., a period of 0.5 h to 4 h) would produce a DC value of ERK* of 0.11–0.16 and an ERK* maximum peak value close to 1. The peak of such an oscillation would depend on the precise input frequency but could persist between 15 min and 2.5 h.

Calculations were also made on the NGF-triggered ERK* responses. For the pulsed inputs (both rectangular and triangular), the ERK* kinetics were remarkably similar to those triggered by EGF. The key difference between EGF and NGF with respect to periodic stimulation was that the ERK* average value was consistently larger with NGF as compared with EGF (see Figure 4 for average ERK* values), a likely result of the positive feedback loop in NGF, which was not present in the EGF circuit.

## 4. Discussion

The advancements in optogenetics and microfluidics have made possible the exciting new branch of synthetic biology, where inputs to cells can be controlled in space and time. While in some instances the goal is to replicate normal physiology (e.g., calcium oscillations), in other cases, the use of synthetic, non-physiological inputs provides information on how cells sense and interpret dynamic environments. Several biological studies indicate that ERK dynamics, whether transient or sustained or wave-like or oscillatory, appear to be important in dictating cell fate in different biological model systems (from cells to embryos) [8,9,10,11,12,13,14,15,16,17,18,19,20,21,22,23,24].

Here, we analyzed a model of the ERK pathway, a pathway that can be triggered externally by growth factors, light or electronically. We calculated the transfer function for the conversion of EGF sinusoids into ERK activation sinusoids. Interestingly, the transfer functions depended on average receptor occupancy, with low to moderate occupancy producing the highest EGF-triggered ERK activation fluctuations. We showed that ERK activation amplitude, shape and duration depended on EGF input pulse high, pulse shape and pulse frequency. We make the new prediction that low frequency, low dose sinusoidal EGF inputs will produce high levels of activated ERK for extended periods. This prediction awaits experimental verification.

The relationship between ERK activation and cell fate is opaque and it is not yet possible to make definitive conclusions regarding EGF input dynamics, ERK dynamics and cell fate. Nevertheless, it is tempting to discuss how EGF input dynamics is hypothesized to influence cell fate (through ERK activation) based on different models of ERK activation and cell fates. There are several models linking ERK activity dynamics to cell fates including sustained-versus-transient [3,8,9,10], the frequency/and/or magnitudes of oscillations [4,11,12,13,14,15], time-integrated activities [16,17,18,19,20] and signals at specific time points [21].

Regarding the sustained-versus-transient model and rectangular input pulses, too infrequent pulses are likely to be seen as a transient decay of the signal, while attempting to re-trigger the signal with very high frequency will also produce very small oscillations (and to the signaling apparatus may appear as a step function input), so qualitatively we would expect a band-pass like filter response to short fixed-width periodic pulses. Use of oscillatory inputs may not be the best way to test transient versus sustained features of the ERK activity. Using exponential ramps for the input should be able to convert a transient response into a sustained response.

If the frequency and/or magnitudes of oscillations are important then the Bode magnitude plot proves very useful. If only the magnitude of oscillations was important then we would predict a decrease in differentiation with increasing frequency, since the high frequency edge of the Bode plots show a decrease in amplitude modulation with increasing frequency, i.e., low-pass filtering. If both frequency and modulation were important, we could see that frequency increases with the input frequency, but the change in modulation with frequency depends on what part of the Bode plot one is operating in. The fate response should increase with frequency in the constant modulation region of the plot, but as frequency continues to increase beyond the breakpoint, the modulation will decrease in proportion to the inverse fifth power of the frequency, and thus reduce the differentiation observed. For the frequency and modulation hypothesis, we qualitatively predict a band-pass filtering behavior.

If time-integrated activities were linked to the differentiation of the cells, then sinusoidal inputs and triangular pulses (with equal on–off periods) should produce integrated ERK* outputs that are independent of frequency. This is because the integral of a sine wave over one cycle is equal to the DC value. With rectangular pulses and at low EGF amplitude the integrated ERK activity will be very low for low frequency pulses but increase with increasing frequency, i.e., a high-pass filter. Persistence of the on-state is another model for cell fate determination. For a low-pass-type filter, for the EGF to ERK fluctuations determined, persistence above a threshold usually generates a band-pass-like filter for EGF to differentiate. The exact relationship depends critically on the predetermined threshold level.

Finally, some authors have produced models showing that the activated level of ERK at a particular time after stimulation is correlated with cell fate (along with some other activated molecules) [21]. If we interpret time after stimulation as time after peak input (in a periodically driven system), then the amount of activated ERK after a particular time delay will depend on the frequency of the input, and on the modulation and the phase of the output. For example, if the signal at time zero was required, then the output signal is the real part of the transfer function, i.e., mcos(φ), where m is the modulation and φ is the phase. If we want the output value at some constant time later [6], we can multiply the real part of the transfer function by exp(-t_d_s), where t_d_ is the (fixed) time delay and s is the complex frequency (or add to the phase angle by an amount ωt_d_).

From experiments published in the literature, EGF-triggered differentiation appears to be band-pass-filter-like, with too infrequent or two frequent rectangular fixed-width pulses producing low differentiation extents and medium frequency inputs producing robust differentiation [4]. We also observed small amounts of differentiation using triangular waves of EGF, which resembled band-pass-like processing (unpublished results). Because the definition of differentiation is complex and involves use of fixed time point data, it is difficult to compare the two results quantitatively. However, the band-pass processing behavior observed for the triangular inputs suggests that the total integrated ERK activity may not be a factor in determining frequency-dependent differentiation responses, at least in the PC12 cells used in the two cited studies.

Whether cells actually respond to the parameters of the output as hypothesized above requires being able to tailor-make ERK* outputs with different output dynamics (shape, amplitude, frequency). Because the ERK* output is a function of the input and the transfer function, knowing the latter should enable the design of experiments to generate ERK* responses that can test the above hypotheses.

## 5. Limitations of Our Study

We have taken a toy model of a signaling system from the literature and studied it. Models are useful for explaining experiments and for predicting new ones but are themselves limited because they do not consider all of the interactions in the system under study. Our results generated from the model will also share these limitations.

We have employed a control theory type view of cell signaling as a complement to other approaches for exploring input–output relationships. The limitations of transfer functions are well-understood; they are designed to work under small perturbations around an equilibrium point. Thus, our analysis and calculations apply to periodic inputs in the linear regime. For the system under study this refers to low receptor occupancy, a situation that corresponds to physiological environments (at least for the EGF receptor).

The question of biological relevance is also a potential limitation. Traditionally, experiments are performed to mimic biology, i.e., constant physiological concentration of growth factor. Dynamically and in a cell culture setting, the growth factor input is a step-function in time, from zero at time zero to some constant concentration at an instant after time zero. The transfer function refers to the Laplace transform of the output under conditions of an impulse in the growth factor concentration, not a step function. Furthermore, with the oscillatory inputs examined here, it is not known when growth factors undergo concentration oscillations in biological settings, if at all. Thus, the use of synthetic inputs is designed to trick the cell into doing something it normally would not do and to test the limits of the system.

Finally, we have used a model where external forcing leads to the forcing of ERK activation. However, there are reported instances where oscillations of ERK activation occur in the presence of constant EGF, i.e., the nucleus–cytoplasmic oscillations of activated ERK by the Wiley group [22]. For the PC12 cells, Ryu et al. [4] did not report sustained oscillations of ERK activity after a pulse or step of EGF; thus, our model is consistent with that report. It would be interesting to change the parameters and compartments of the current model to see where fixed concentration EGF inputs can produce ERK activity oscillations and whether these could be entrained externally. Recent work indicates the ability to entrain these oscillations with AC electrical currents [24].

## 6. Conclusions

Frequency domain analysis brings us closer to a functional understanding of how biochemical cascades function in a complex and rapidly changing environment. Our analysis indicates some non-intuitive features of the ERK pathway. Our prediction is that low doses of growth factors delivered over periods of hours will lead to the largest dynamic ERK activation responses. This counters the simple view that receptors need to be fully occupied to generate optimal responses and that biological circuits are designed to respond to rapidly changing environments.

## Figures and Tables

**Table 1:** Parameters used in the ERK pathway model and taken from [4].

Constant	Value	Units	Constant	Value	Units	Constant	Value	Units
k1R	0.5/60	$s^{-1}$	kd1R	0.5/60	$s^{-1}$	PtaseR	1	unitless
k 2	2/60	$s^{-1}$	K 2	1	unitless	kd 2	0.25/60	$s^{-1}$
D 2	0.1	unitless	k 3 F	0.0286/60	$s^{-1}$	K 3	0.01	unitless
K3R	0.85	unitless	kd 3	0.0057/60	$s^{-1}$	D 3	0.5	unitless
PtaseNFB	1	unitless	k 4	2/60	$s^{-1}$	K 4	1	unitless
kd4	0.5/60	$s^{-1}$	D 4	1	unitless	PtaseMEK	1	unitless
k 5	10/60	$s^{-1}$	K 5	1	unitless	kd5	3.75/60	$s^{-1}$
D5	1	unitless	KNFB	0.05	unitless	PtaseRaf	1	unitless
kPFB	0/60 (EGF) 0.75/60 (NGF)	$s^{-1}$	KPFB	0.01	unitless	k 6 R	40/60	$s^{-1}$
K6	1	unitless	kd6	7.5/60	$s^{-1}$	D 6	1	unitless
GAP	1	unitless	k 7	0.1/60	$s^{-1}$	K 7	0.1	unitless
kd7	0.005/60	$s^{-1}$	D7	0.1	unitless	PtasePFB	1	unitless
duspbasal	1	unitless	duspind	6	unitless	Kdusp	0.1	unitless
Tdusp	90∗60	s	TDUSP	90∗60	s			

## Data Availability

Source code and data are available at: http://github.com/nguyenhntran/ERKpaper2025 (accessed on 2 April 2025). Data used to generate the figures can be found in the “Figures” directory, with subfolders corresponding to figure numbers as referenced throughout the manuscript. Source code and datasets are located in the “SourceCode” directory. The structure of key directories is as follows: ERKpathway_EGFactivation: Simulations of the ERK pathway under EGF stimulation; General_TransFunc: Computation of Jacobians describing pathway dynamics at equilibrium; RectangularPulseTrain: Simulations of ERK* responses to rectangular EGF pulse inputs; *Equilibria*: Calculation of steady-state concentrations under pulse train mean forcing; *TransferFunction*: Derivation of pathway transfer functions at the computed equilibria; *Transformation*: Simulation of ERK* dynamics using the derived transfer functions; TriangularPulseTrain: Simulations of ERK* responses to triangular EGF pulse inputs; *Equilibria*: Calculation of steady-state concentrations under pulse train mean forcing; *TransferFunction*: Derivation of pathway transfer functions at the computed equilibria; *Transformation*: Simulation of ERK* dynamics using the derived transfer functions. ERKpathway_NGFactivation: Simulations of the ERK pathway under NGF stimulation; General_TransFunc: Computation of Jacobians describing pathway dynamics at equilibrium; RectangularPulseTrain: Simulations of ERK* responses to rectangular NGF pulse inputs; *Equilibria*: Calculation of steady-state concentrations under pulse train mean forcing; *TransferFunction*: Derivation of pathway transfer functions at the computed equilibria; *Transformation*: Simulation of ERK* dynamics using the derived transfer functions; TriangularPulseTrain: Simulations of ERK* responses to triangular NGF pulse inputs; *Equilibria*: Calculation of steady-state concentrations under pulse train mean forcing; *TransferFunction*: Derivation of pathway transfer functions at the computed equilibria; *Transformation*: Simulation of ERK* dynamics using the derived transfer functions.
